# Association of endocrine immune-related adverse events with progression-free survival in advanced non-small cell lung cancer treated with PD-1/PD-L1 inhibitors with or without anlotinib

**DOI:** 10.3389/fonc.2026.1701750

**Published:** 2026-03-02

**Authors:** Furong Sun, Yuzhu Gao, Xiaoyan Zheng, Jie Hua, Shouzhen Liu, Dong Cao, Jianping Zhou

**Affiliations:** 1 Department of Respiratory and Critical Care Medicine, Zhoushan Branch, Ruijin Hospital, Shanghai Jiao Tong University School of Medicine, Zhoushan, China; 2 School of Medical Information Engineering, Guangzhou University of Chinese Medicine, Guangzhou, China; 3 Department of Respiratory and Critical Care Medicine, Ruijin Hospital, Shanghai Jiao Tong University School of Medicine, Shanghai, China; 4 Institute of Respiratory Diseases, Shanghai Jiao Tong University School of Medicine, Shanghai, China

**Keywords:** anlotinib, endocrine immune-related adverse events, non-small-cell lung cancer, PD-1/PD-L1 inhibitors, progression-free survival

## Abstract

**Introduction:**

Endocrine immune-related adverse events (irAEs) are frequently observed during PD-1/PD-L1 therapy and may indicate active immune engagement during treatment. However, it remains uncertain whether this association persists in regimens incorporating anlotinib.

**Methods:**

We retrospectively analyzed 77 consecutive patients with advanced NSCLC who received PD-1/PD-L1 inhibitors plus platinum-based chemotherapy with (n = 17) or without (n = 60) anlotinib. Endocrine irAEs were defined according to the CTCAE v5.0 using assay-specific thresholds. To address the immortal-time bias, we applied prespecified 12- and 24-week landmark analyses and a time-dependent Cox model. Effect estimates were presented with 95% confidence intervals.

**Results:**

Endocrine irAEs were predominantly grades 1–2 and occurred later in patients treated with anlotinib (median onset 12 vs. 9 weeks). In the 12- and 24-week landmark analyses, where irAE status was determined at the landmark, endocrine irAEs were not significantly associated with PFS in the overall cohort (12-week HR 1.23, 95% CI 0.70–2.17; 24-week HR 1.27, 95% CI 0.67–2.43). Similarly, a time-dependent Cox model treating endocrine irAEs as time-varying covariates did not demonstrate a protective effect (HR 2.38, 95% CI 1.43–3.94). Adjusted comparisons indicated no meaningful PFS difference between treatment regimens, and the findings from the anlotinib subgroup (n = 17) were exploratory.

**Conclusion:**

In this single-center cohort, endocrine irAEs functioned as dynamic on-treatment indicators but did not confer a clear PFS advantage after bias-aware modeling. Given the limited sample size, these findings are exploratory and require further prospective validation.

## 1 Introduction

Immune checkpoint inhibitors (ICIs) targeting the PD-1/PD-L1 axis have transformed first-line therapy for advanced NSCLC. The 5-year update of *KEYNOTE-024* reported an overall survival (OS) rate of 31.9% with pembrolizumab monotherapy, nearly doubling that achieved with platinum doublet chemotherapy (1). Similarly, *CheckMate-227* demonstrated a 24% 5-year OS with nivolumab plus ipilimumab compared to 13% with chemotherapy (2). Nevertheless, only a minority of unselected patients experience durable benefits, underscoring the need for *dynamic*, on-treatment biomarkers that can complement baseline PD-L1 expression or tumor mutational burden (3).

Immune-related adverse events (irAEs) have emerged as pharmacodynamic, treatment-embedded indicators of ICI activity in patients with cancer. Endocrine toxicities, including thyroid dysfunction, hypophysitis, and adrenal insufficiency, are among the most frequent irAEs and are readily detected in routine clinical practice (4). Meta-analyses and real-world studies have reported associations between early low-grade irAEs and improved survival outcomes (5–7). Beyond epidemiology, irAEs may more accurately reflect contemporaneous immune activation than baseline PD-L1, which is spatially and temporally heterogeneous and subject to metabolic remodeling (e.g., PD-L1 lactylation) (8). Large real-world cohorts further highlight the clinical heterogeneity and *time-dependent* nature of irAEs, emphasizing the importance of analytic approaches that appropriately address time-related bias (9). Recent registry-based analyses have also examined rarer irAEs, such as incident non-infectious uveitis after ICI exposure, offering methodological parallels and a comparative context for irAE research (10). In parallel, host–microbial nutrient pathways are increasingly recognized as modulators of antitumor immunity and autoimmune thresholds, providing mechanistic support for the use of irAEs as dynamic, treatment-integrated biomarkers alongside PD-L1 (11).

In China, ICIs are increasingly administered in combination with the multi-target anti-angiogenic tyrosine kinase inhibitor (TKI) anlotinib. Contemporary reviews of ICI combination strategies highlight the expanding rationale for pairing checkpoint blockade with targeted and microenvironment-modulating agents, providing context for anti-angiogenic/ICI regimens, such as anlotinib combinations (12). Feasibility studies have reported objective response rates of 28.4%–47.5% and median progression-free survival (PFS) of 6–8 months without prohibitive toxicity (13–15). However, the prognostic relevance of endocrine irAEs in such regimens has not been established.

To address this gap, we retrospectively evaluated the incidence, temporal characteristics, and prognostic impact of endocrine irAEs on PFS in patients with advanced NSCLC receiving PD-1/PD-L1 inhibitors with or without anlotinib, providing hypothesis-generating evidence for subsequent prospective multicenter validation.

## 2 Materials and methods

### 2.1 Study design

This retrospective, single-center cohort study was conducted at the Department of Respiratory and Critical Care Medicine, Zhoushan Branch, Ruijin Hospital, Shanghai Jiao Tong University School of Medicine. This study investigated the association between endocrine immune-related adverse events (irAEs) and progression-free survival (PFS) in patients with advanced NSCLC treated with PD-1/PD-L1 inhibitors in combination with platinum-based chemotherapy, with or without anlotinib. The study period spanned July 2018 to July 2025. The study protocol was approved by the institutional ethics committee, and the requirement for informed consent was waived owing to its retrospective design.

### 2.2 Inclusion and exclusion criteria

The inclusion criteria were as follows: (a) age ≥18 years; (b) histologically or cytologically confirmed stage IIIB/IV or recurrent NSCLC; (c) receipt of at least two cycles of a PD-1/PD-L1 inhibitor plus platinum-based chemotherapy, with or without anlotinib; (d) availability of complete clinical, radiological, and laboratory data, including thyroid and adrenal function tests; and (e) Eastern Cooperative Oncology Group (ECOG) performance status of 0–1.

The exclusion criteria were as follows: (a) small cell lung cancer or other non-NSCLC histologies; (b) incomplete follow-up or missing outcome data; (c) pre-existing endocrine disorders that could confound irAE assessment; (d) participation in concurrent interventional trials; and (e) ECOG performance status ≥2.

**Figure 1:**
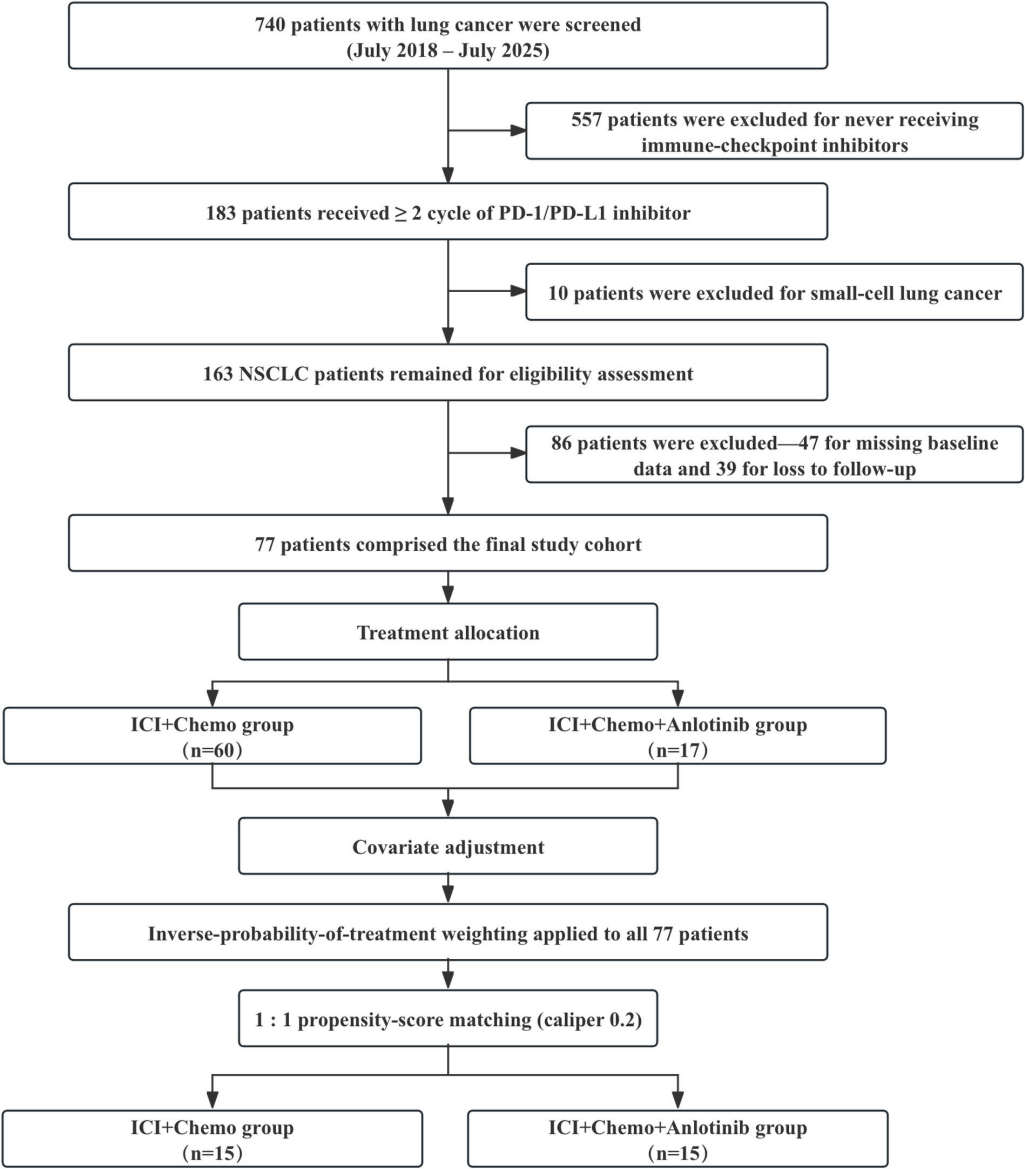
Flow diagram of patient screening, exclusion and allocation.

A total of 77 patients met the eligibility criteria and were included in the final analyses. Among them, 60 received a PD-1/PD-L1 inhibitor plus platinum-based chemotherapy (ICI + Chemo group), and 17 received the same regimen with the addition of anlotinib (ICI + Chemo + Anlotinib group). A flowchart summarizing the patient selection process is presented in **Figure 1**.

### 2.3 Data collection

Clinical data were extracted from the electronic medical records. The baseline variables included age, sex, smoking history, histological subtype, and TNM stage. Treatment-related information included the type of immunotherapy, chemotherapy backbone, anlotinib use, and treatment duration. In the ICI + Chemo + Anlotinib arm, anlotinib was administered orally at a starting dose of 12 or 10 mg once daily on days 1–14 of each 21-day cycle, at the treating physician’s discretion, until disease progression or unacceptable toxicity; dose reductions were permitted according to tolerability (**Supplementary Table S1**).

Endocrine irAEs were adjudicated and graded according to the CTCAE v5.0. Overt thyroid dysfunction required concordant abnormalities in TSH and FT4, whereas secondary adrenal insufficiency required low morning cortisol levels accompanied by an inappropriately low or normal ACTH level and/or an insufficient response to 250 µg cosyntropin. In accordance with institutional policy, thyroid (TSH/FT4) and adrenal (cortisol/ACTH) panels were performed at baseline and at each ICI treatment cycle (approximately every 3 weeks). The assay-specific thresholds are detailed in **Supplementary Methods S1** and **Supplementary Table S2**.

Radiologic response was assessed every 6–8 weeks using CT or MRI and was evaluated according to the RECIST version 1.1. The primary endpoint was PFS, defined as the interval from treatment initiation to documented disease progression or death. Patients without progression were censored on the date of their last imaging assessment.

### 2.4 Statistical analysis

The baseline characteristics were summarized descriptively. Continuous variables are reported as medians with interquartile ranges (IQRs) and were compared using the Mann–Whitney *U* test. Categorical variables were compared using the *χ*^2^ test or Fisher’s exact test.

Between-regimen comparisons (ICI + Chemo vs. ICI + Chemo + Anlotinib) targeted the average treatment effect and were adjusted using stabilized inverse probability of treatment weighting (IPTW). Propensity scores were estimated using logistic regression, including age, sex, BMI, smoking status, histology, and disease stage. Covariate balance was assessed using weighted standardized mean differences (SMDs), with |SMD| *<*0.15 considered acceptable. Weighted Kaplan–Meier curves and robust Cox models were used to estimate the hazard ratios (HRs) and 95% confidence intervals (CIs).

To address the immortal-time bias in evaluating endocrine irAEs and PFS, we performed prespecified landmark analyses at 12 and 24 weeks, chosen *a priori* to align with the typical onset window of endocrine irAEs and with our institutional imaging schedule (first restaging at 12 weeks), thereby capturing both early and intermediate on-treatment exposure. Patients who were event-free at each landmark were included; the follow-up time was reset to the landmark, and endocrine irAE status was defined using information up to that point. A complementary time-dependent Cox model treated endocrine irAE as a time-varying covariate in the counting process format and adjusted for the same baseline covariates. Because of sparse events, regimen-stratified analyses were regarded as exploratory, and Firth-penalized Cox models were used when quasi-separation was suspected.

As exploratory prediction tools, we fitted L1-penalized Cox models for PFS and logistic regression models for endocrine irAE occurrence, evaluating performance using cross-validated C-indices and ROC AUC. Details of the model implementation and additional sensitivity analyses, including 1:1 propensity score matching, are provided in the **Supplementary Methods**. Given the modest overall sample size, particularly the small anlotinib arm, all subgroup and machine learning analyses were interpreted descriptively, whereas the primary inference relied on the bias-aware landmark and time-dependent Cox models. All analyses were conducted in Python 3.11 using lifelines, pandas, statsmodels, and scikit-learn. A two-sided p <0.05 was considered statistically significant.

## 3 Results

### 3.1 Patient characteristics

A total of 77 patients with advanced NSCLC were included in the analysis; 17 patients (22.1%) received ICI + Chemo + Anlotinib and 60 (77.9%) received ICI + Chemo. The baseline characteristics were generally comparable between the groups (**Table 1**). The median age was 66 years, and most patients were male and were current or former smokers. The distributions of histology, disease stage, line of therapy, baseline brain metastases, corticosteroid exposure, prior TKI treatment, chemotherapy backbone, and treatment intensity (cycles ≥4 within 12 weeks) did not differ significantly between the arms (all *P >*0.05). Baseline PD-L1 tumor proportion score (TPS) and oncogenic driver status were available for a minority of patients and are summarized by treatment arm in **Supplementary Table S4**.

**Table 1:** Baseline characteristics of patients treated with or without Anlotinib.

Characteristic	Overall (n = 77)	ICI+Chemo+Anlotinib (n = 17)	ICI+Chemo (n = 60)	*P* value
Age, median (range), years				
	66 (49–79)	64 (49–78)	67 (49–79)	0.57
Sex, n (%)				
Male	62 (80.5)	13 (76.5)	49 (81.7)	0.73
Female	15 (19.5)	4 (23.5)	11 (18.3)	
Smoking status, n (%)				
Current/Former	72 (93.5)	15 (88.2)	57 (95.0)	0.30
Never	5 (6.5)	2 (11.8)	3 (5.0)	
Histological features, n (%)				
Adenocarcinoma	34 (44.2)	8 (47.1)	26 (43.3)	0.79
Non-adenocarcinoma	43 (55.8)	9 (52.9)	34 (56.7)	
Stage, n (%)				
III	34 (44.2)	6 (35.3)	28 (46.7)	0.41
IV	43 (55.8)	11 (64.7)	32 (53.3)	
Line of therapy, n (%)				
1	53 (68.8)	9 (52.9)	44 (73.3)	0.17
2	9 (11.7)	2 (11.8)	7 (11.7)	
≥3	15 (19.5)	6 (35.3)	9 (15.0)	
Brain metastases at baseline, n (%)				
Yes	14 (18.2)	6 (35.3)	8 (13.3)	0.07
No	63 (81.8)	11 (64.7)	52 (86.7)	
Baseline corticosteroid ≥10 mg (within 14 d), n (%)				
Yes	12 (15.6)	3 (17.6)	9 (15.0)	0.72
No	65 (84.4)	14 (82.4)	51 (85.0)	
Prior TKI exposure, n (%)				
Yes	12 (15.6)	3 (17.6)	9 (15.0)	0.72
No	65 (84.4)	14 (82.4)	51 (85.0)	
Chemotherapy backbone, n (%)				
pemetrexed	25 (32.5)	6 (35.3)	19 (31.7)	0.53
non-pemetrexed	52 (67.5)	11 (64.7)	41 (68.3)	
Treatment cycles ≥4 within 12 weeks, n (%)				
Yes	60 (77.9)	16 (94.1)	44 (73.3)	0.10
No	17 (22.1)	1 (5.9)	16 (26.7)	

### 3.2 Incidence and characteristics of endocrine irAEs

Endocrine irAEs occurred in 11 of 17 patients (64.7%) in the ICI + Chemo + Anlotinib group and in 36 of 60 patients (60.0%) in the ICI + Chemo group. Baseline characteristics were broadly similar between patients with and without endocrine irAEs, except that in the ICI + Chemo arm, stage IV disease was more frequent among those who developed endocrine irAEs (72.2% vs. 25.0%, *P <*0.001; **Supplementary Table S3**).

Endocrine irAEs were predominantly grades 1–2 across both arms. The median onset was later in the ICI + Chemo + Anlotinib group (12.0 weeks) than in the ICI + Chemo group (9.0 weeks). Thyroid dysfunction was the most common event, whereas adrenal insufficiency was infrequent. The detailed incidence, grading, and onset distributions are summarized in **Table 2**.

**Table:** 2Incidence, severity and onset of endocrine irAEs in the two treatment arms.

Characteristic	ICI + Chemo + Anlotinib	ICI + Chemo	RD, pp [95% CI]	*P* value
Endocrine irAEs (any)	11/17 (64.7%)	36/60 (60.0%)	4.7 [−30.1, 35.3]	0.785
Thyroid dysfunction (any)	10/17 (58.8%)	32/60 (53.3%)	5.5 [−29.4, 37.5]	0.786
Overt hyperthyroidism	5/17 (29.4%)	20/60 (33.3%)	−3.9 [−32.7, 30.4]	1.000
Overt hypothyroidism	4/17 (23.5%)	10/60 (16.7%)	6.9 [−18.5, 37.9]	0.496
Subclinical hyperthyroidism	1/17 (5.9%)	2/60 (3.3%)	2.5 [−10.3, 26.1]	0.532
Adrenal insufficiency (any)	1/17 (5.9%)	5/60 (8.3%)	−2.5 [−17.0, 23.4]	1.000
Secondary adrenal insufficiency	1/17 (5.9%)	4/60 (6.7%)	−0.8 [−14.9, 24.4]	1.000
Grade 3–4 endocrine irAE	0/17 (0.0%)	1/60 (1.7%)	−1.7 [−8.9, 18.1]	1.000

*P*roportions are shown as n/N (%). Two-sided P values are from Fisher’s exact tests. Risk differences (RD, percentage points) and 95% CIs were computed using the Newcombe–Wilson method (without continuity correction).

After inverse probability of treatment weighting, all prespecified covariates achieved an acceptable balance (weighted absolute SMDs <0.15; most <0.10), and the two arms demonstrated good overlap in propensity score distributions (**Supplementary Figure S1**).

### 3.3 Progression-free survival by endocrine irAE status

**Figure 2:**
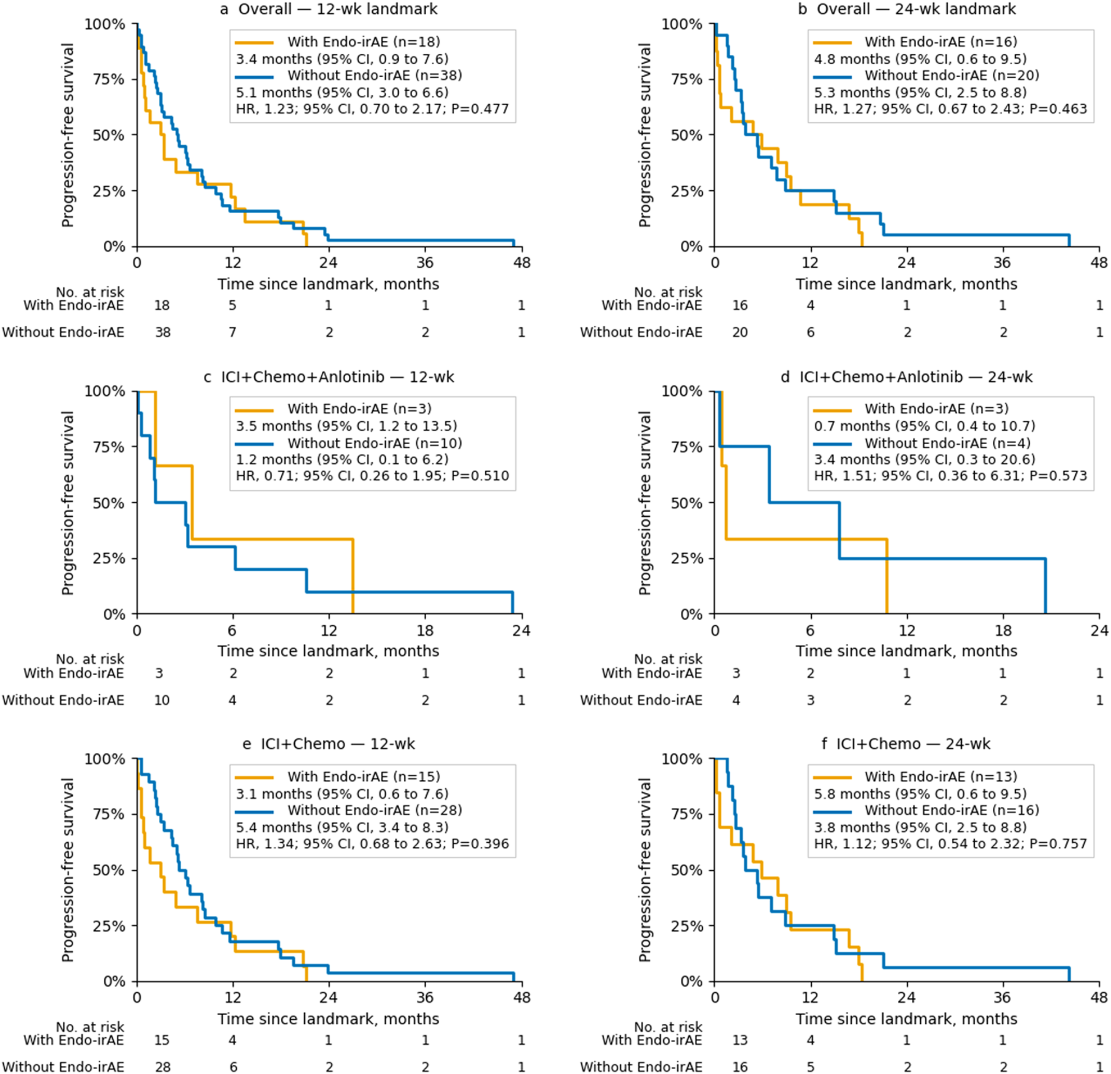
PFS by endocrine irAE status from landmark analyses. **(A)** Overall—12-week landmark; **(B)** Overall—24-week landmark; **(C)** ICI + Chemo + Anlotinib—12-week landmark; **(D)** ICI + Chemo + Anlotinib—24-week landmark; **(E)** ICI + Chemo—12-week landmark; and **(F)** ICI + Chemo—24-week landmark. Hazard ratios (HRs) and two-sided *p*-values from Cox models are annotated within each panel.

In strict landmark analyses, endocrine irAEs were not significantly associated with PFS in the overall cohort. At both the 12- and 24-week landmarks, the landmark-defined irAE status did not confer a PFS advantage, and the hazard ratios were close to unity (**Figure 2**). Specifically, the 12- and 24-week Cox models yielded HRs of 1.23 and 1.27, respectively, with 95% confidence intervals crossing 1.0.

Stratified analyses by treatment regimen were limited by small sample sizes and sparse events, particularly in the ICI + Chemo + Anlotinib arm. The estimates were imprecise and did not demonstrate consistent trends across landmarks (**Figure 2**). Therefore, these subgroup results should be interpreted as exploratory.

In a complementary time-dependent Cox model treating endocrine irAEs as a time-varying exposure and adjusting for baseline covariates, the onset of an endocrine irAE was associated with a higher instantaneous hazard of progression (HR = 2.38, 95% CI, 1.43–3.94). This finding aligns with landmark analyses, indicating that endocrine irAEs did not act as protective biomarkers in this cohort.

Taken together, endocrine irAEs did not demonstrate a robust association with PFS in either the overall population or treatment-specific subgroups across both bias-aware approaches.

Using 1:1 nearest-neighbor matching on the logit of the propensity score (caliper = 0.2 × SD), 15 matched pairs were generated. In the matched cohort, there was no significant difference in PFS between regimens (HR 1.14, 95% CI 0.55–2.35; *P* = 0.730), consistent with the IPTW main analysis, which showed no clear between-regimen effect.

### 3.4 Exploratory machine learning

Exploratory predictive analyses were conducted to complement the association models. A baseline LASSO–Cox model using only pre-treatment clinical variables showed limited discriminative ability and modest calibration. Incorporating endocrine irAE status into a 12-week landmark model modestly improved performance, although the overall predictive ability remained weak. Across models, smoking status, sex, and stage contributed the largest coefficients, whereas the treatment arm and other baseline features had smaller effects.

An L1-penalized logistic regression model predicting endocrine irAE occurrence from baseline variables demonstrated moderate discrimination. Detailed performance metrics and coefficient plots are presented in **Supplementary Figure S2**.

These exploratory models, constrained by sample size, highlight the limited prognostic value of static baseline features and the potential incremental information provided by time-updated on-treatment signals.

## 4 Discussion

In this single-center retrospective cohort study of 77 patients with advanced NSCLC treated with PD-1/PD-L1 inhibitors plus platinum chemotherapy with or without anlotinib, endocrine immune-related adverse events (irAEs) were common, predominantly grades 1–2, and occurred later in anlotinib-containing regimens (median onset 12.0 vs. 9.0 weeks). However, in bias-aware analyses, including prespecified 12- and 24-week landmarks and a time-dependent Cox model, endocrine irAEs were not associated with a clear progression-free survival advantage in the overall cohort. The landmark analyses yielded HRs of 1.23 and 1.27 at 12 and 24 weeks, respectively, and the time-dependent model indicated a higher instantaneous hazard of progression after irAE onset (HR 2.38, 95% CI 1.43–3.94). These findings suggest that endocrine irAEs in this setting may function primarily as dynamic on-treatment markers of immune activation and treatment exposure rather than as reliable surrogate indicators of benefit. Subgroup estimates within the ICI + Chemo + Anlotinib arm were highly imprecise due to small numbers and should be interpreted with caution. Notably, only one grade 3–4 endocrine event was observed, indicating that endocrine toxicities were generally manageable with routine monitoring.

These results refine and contextualize previous evidence linking endocrine irAEs, particularly early, low-grade events, to favorable clinical outcomes. In a real-world lung cancer cohort (*n* = 983), mostly low-grade endocrine irAEs emerging at approximately 4.1 months were associated with prolonged progression-free survival (10.7 vs. 3.8 months; HR 0.50) and overall survival (31.6 vs. 10.8 months; HR 0.36) (6). A meta-analysis across tumor types similarly reported improved survival among patients experiencing early, low-grade irAEs (pooled HR 0.42 for progression-free survival; HR 0.57 for overall survival), while also demonstrating the increased risk of endocrine toxicities with PD-1/PD-L1 blockade and underscoring the need for systematic monitoring (16). In contrast, when the immortal-time bias was explicitly addressed in our smaller single-center cohort, the apparent survival advantage was attenuated and no longer robust. Although wide confidence intervals do not exclude the possibility of modest benefit or harm, the overall direction and magnitude of our bias-aware estimates highlight that the associations between irAEs and outcomes can vary across clinical settings and are highly sensitive to study design, assessment intervals, and analytic methods.

The anlotinib-related observations in our cohort should be interpreted in the context of the limited sample size and the absence of a clear between-regimen PFS difference after weighting and matching analyses. Retrospective studies in previously treated advanced NSCLC have reported a median PFS of 6.5 months and a median OS of 15.8 months for anlotinib combined with PD-1 blockade (13). A real-world propensity-matched analysis further suggested that adding anlotinib to ICIs plus platinum chemotherapy could prolong PFS compared with ICIs plus platinum alone (median 7.76 vs. 2.33 months; HR 0.23, *P* = 0.012) (14). Our findings neither confirm nor contradict these potential regimen-level advantages but indicate that within this cohort, endocrine irAEs did not mediate a measurable PFS benefit.

Mechanistically, anti-angiogenic tyrosine kinase inhibition may modulate immune activation in ways that influence irAE development. Anlotinib has been shown to transiently normalize aberrant tumor vasculature, enhance endothelial adhesion programs, and upregulate CXCL9/10–CXCR3 chemokine signaling, thereby improving CD8^+^ T-cell recruitment and retention. Preclinical models have demonstrated prolonged vascular normalization and synergy with PD-1 blockade (17). Clinically, pooled analyses have reported increased rates of specific treatment-related toxicities, including hypothyroidism, when VEGF/angiogenesis inhibitors are combined with ICIs versus ICIs alone (18). These data support a biologically coherent link between anlotinib-enhanced immune activation and the propensity for endocrine irAEs, reinforcing the rationale for proactive endocrine monitoring and the application of time-aware analytic methods in anti-angiogenic/ICI combination therapy.

The delayed onset of endocrine irAEs in anlotinib-containing regimens in our study further suggests the regimen-dependent kinetics of immune activation. Combination therapy may gradually remodel the vascular and immune microenvironments, shifting the timing of endocrine toxicities while maintaining antitumor immune enhancement. Notably, intensified immune activation, as seen with combination checkpoint blockade, is associated with a higher endocrine irAE risk, particularly hypophysitis (19), indicating that endocrine toxicity may increase in parallel with immune potentiation.

The methodological strength of this study was the use of prespecified 12- and 24-week landmark analyses together with a time-dependent Cox model, allowing explicit mitigation of the immortal time bias and proper handling of the time-varying nature of endocrine irAEs. Without such approaches, patients must remain event-free long enough to develop an irAE, leading to spurious associations between irAE occurrence and improved survival outcomes. Prior meta-research has shown that effect sizes often attenuate substantially when the immortal time bias is addressed (20). Recent meta-epidemiological evidence further suggests that immortal-time bias is prevalent across survival meta-analyses and can materially influence pooled effect estimates and the vulnerability of evidence, supporting the routine use of time-robust designs in irAE-outcome research (21). Consistent with this, our strict landmark models yielded HRs near 1.0 at both time points, and the time-dependent model indicated a higher risk of progression after irAE onset. These bias-aware estimates demonstrate how survival advantages inferred from less rigorous designs may diminish in smaller real-world cohorts and underscore the importance of appropriately modeling time-dependent exposures.

The endocrine toxicity patterns in our study were consistent with those reported in prior NSCLC cohorts: thyroid dysfunction predominated, adrenal insufficiency was infrequent, and nearly all events were grade 1–2 (22). Multiple studies have associated thyroid irAEs with improved NSCLC outcomes (14). Routine surveillance of TSH/FT4 and cortisol/ACTH, along with timely hormone replacement, when necessary, can support the safe continuation of ICI therapy (23) and likely contributed to the low incidence of high-grade events in our study cohort.

Our exploratory modeling results further support the concept that endocrine irAEs are dynamic on-treatment indicators rather than fixed baseline predictors. Incorporating endocrine irAE status into a 12 week landmark LASSO–Cox model modestly improved discrimination relative to a baseline-only model, while an L1-penalized logistic model predicted endocrine irAE occurrence with moderate accuracy. Although limited by sample size and not intended for causal inference, these analyses highlight the modest prognostic value of static baseline features and the potential incremental information conveyed by time-updated clinical signals. Future work may benefit from explainable AI-enabled clinical decision support frameworks that emphasize interpretability, clinician trust, and usability when developing transparent, time-updated models for irAE risk stratification and outcome prediction (24).

This study has several limitations. This retrospective, single-center cohort study, particularly the small anlotinib subgroup, limited the statistical power and resulted in imprecise estimates, even with propensity-based adjustment and sensitivity analyses. Because treatment allocation was non-random and accrual spanned seven years, residual confounding (including confounding by indication and calendar-time changes in practice) may remain despite IPTW/PSM and time-aware modeling. Endocrine irAEs were captured through routine laboratory surveillance but may still be subject to some misclassification, and missing PD-L1/TMB together with treatment heterogeneity constrained more granular adjustments. Finally, although landmark and time-dependent approaches mitigate immortal-time bias, landmark analyses condition on being event-free at the landmark and may not fully represent early progressors; the 12- and 24-week time points were prespecified and clinically motivated but were not unique.

Clinically, these findings support proactive endocrine surveillance and conservative management of low-grade irAEs during ICI-based therapy, including regimens that incorporate anlotinib. In our bias-aware analyses, endocrine irAEs functioned more as dynamic, on-treatment indicators of pharmacodynamic immune engagement than as reliable surrogate markers of treatment benefit, and their prognostic implications remain unclear. Prospective multicenter studies with standardized endocrine monitoring and prespecified time-dependent methods are needed to determine how on-treatment signals can be integrated into individualized immunotherapy strategies.

## 5 Conclusion

In this single-center cohort of patients with advanced NSCLC receiving PD-1/PD-L1 inhibitors plus chemotherapy with or without anlotinib, endocrine irAEs were common, generally low-grade, and occurred later in anlotinib-containing regimens. Bias-aware 12- and 24-week landmark analyses and a time-dependent Cox model did not demonstrate a robust progression-free survival advantage associated with endocrine irAEs, and no meaningful PFS difference between regimens was observed at the current sample sizes. Given the retrospective design and modest sample size, particularly in the anlotinib subgroup, these observations should be regarded as hypothesis generating. Prospective multicenter studies with standardized endocrine monitoring and prespecified time-dependent analytic frameworks are necessary to confirm the clinical utility and clarify how endocrine irAEs may be incorporated into personalized chemo-immunotherapy and anti-angiogenic treatment pathways.

## Data Availability

The original contributions presented in the study are included in the article/**Supplementary Material**. Further inquiries can be directed to the corresponding authors.
