# Correction: Association of endocrine immune-related adverse events with progression-free survival in advanced non-small cell lung cancer treated with PD-1/PD-L1 inhibitors with or without anlotinib

**DOI:** 10.3389/fonc.2026.1847088

**Published:** 2026-04-22

**Authors:** Furong Sun, Yuzhu Gao, Xiaoyan Zheng, Jie Hua, Shouzhen Liu, Dong Cao, Jianping Zhou

**Affiliations:** 1 Department of Respiratory and Critical Care Medicine, Zhoushan Branch, Ruijin Hospital, Shanghai Jiao Tong University School of Medicine, Zhoushan, China; 2 School of Medical Information Engineering, Guangzhou University of Chinese Medicine, Guangzhou, China; 3 Department of Respiratory and Critical Care Medicine, Ruijin Hospital, Shanghai Jiao Tong University School of Medicine, Shanghai, China; 4 Institute of Respiratory Diseases, Shanghai Jiao Tong University School of Medicine, Shanghai, China

**Keywords:** anlotinib, endocrine immune-related adverse events, non-small-cell lung cancer, PD-1/PD-L1 inhibitors, progression-free survival

In the published article, file Table 1 was erroneously associated with the published article record as supplementary material. The file has now been removed.

The original version of this article has been updated.

