# Supplementary material for: Association of endocrine immune-related adverse events with progression-free survival in advanced non-small cell lung cancer treated with PD-1/PD-L1 inhibitors with or without anlotinib
Source: Front Oncol. 2026 Mar 2;16:1701750. doi: 10.3389/fonc.2026.1701750 (PMC12989326; doi:10.3389/fonc.2026.1701750)
Supplement: Supplementary file 1 [file DataSheet1.pdf]

1 SUPPLEMENTARY METHODS

1.1 Endocrine irAE ascertainment, grading, and monitoring

Endocrine adverse events (AEs) were graded according to the Common Terminology Criteria for Adverse Events (CTCAE) version 5.0. AEs were adjudicated as immune-related based on temporal patterns and laboratory trajectories (e.g., transient thyrotoxicosis followed by hypothyroidism) after exclusion of alternative causes. Discrepancies were resolved by consensus, with consultation from the endocrinology service when needed. In sensitivity analyses, the endpoint was restricted to overt or treatment-requiring events, defined as those necessitating levothyroxine or glucocorticoid replacement.

Numeric thresholds followed institutional laboratory reference ranges; assay-specific cutoffs are listed in Supplementary Table S1. Briefly, overt hypo- or hyperthyroidism required concordant abnormalities in TSH and FT4. Subclinical thyroid dysfunction was defined by isolated TSH elevation or suppression with normal FT4. Central hypothyroidism required low FT4 with low or inappropriately normal TSH. Secondary adrenal insufficiency required a low 08:00–09:00 serum cortisol with low or inappropriately normal ACTH and/or a subnormal peak cortisol on standard 250 µg cosyntropin testing.

Per institutional policy, thyroid (TSH/FT4) and adrenal (morning cortisol/ACTH) panels were obtained at baseline and with each ICI treatment cycle (approximately every 3 weeks). These tests were embedded in the infusion order sets and thus applied identically in both treatment arms. If an infusion was advanced or delayed for clinical reasons, endocrine tests were performed on the next infusion day; additional symptom-triggered testing was conducted at the treating physician's discretion. This uniform cycle-linked schedule minimized systematic differences in surveillance intensity between arms, although residual deviations are acknowledged as a study limitation.

1.2 Propensity score diagnostics and robustness

Between-regimen comparisons (ICI+Chemo+Anlotinib vs. ICI+Chemo) targeted the average treatment effect (ATE). Propensity scores (PS) were estimated using logistic regression including age, sex, body mass index (BMI), smoking status (ever vs. never), histology (adenocarcinoma vs. non-adenocarcinoma), and disease stage (IV vs. III). Stabilized inverse-probability-of-treatment weights (IPTW) were calculated as $w = P(A)/PS$ for treated patients and $w = [1 - P(A)]/[1 - PS]$ for controls. Weighted Cox models with robust variance estimates were used to compute hazard ratios (HRs) and 95% confidence intervals (CIs).

Observed PS values ranged from 0.125–0.487 in the treated arm and 0.118–0.444 in the control arm. Common-support trimming to the interval [0.125, 0.444] excluded four patients (one treated, three controls). Figure S1 (right panel) shows arm-specific PS histograms demonstrating good overlap.

Stabilized weights were well behaved, without extreme tails. Overall percentiles (minimum, 5th, 25th, median, 75th, 95th, maximum) were 0.454, 0.791, 0.929, 0.989, 1.053, 1.286, and 1.764, respectively. By arm, median weights were 0.951 (treated; 5th–95th percentiles: 0.575–1.625) and 0.989 (control; 5th–95th percentiles: 0.895–1.147). The corresponding Kish effective sample sizes (ESS) were 15.4/17 for treated patients and 59.5/60 for controls (total ESS 74.9/77), indicating minimal loss of information.

Standardized mean differences (SMDs) were computed before and after weighting. All prespecified covariates achieved $|\mathrm{SMD}| < 0.15$ after IPTW (most < 0.10), as shown in the Love plot in Figure S1 (left panel).

Multiple robustness checks were performed. (i) Weight truncation at the 1st/99th and 5th/95th percentiles produced HRs of 1.24 (95% CI 0.71–2.17) and 1.23 (95% CI 0.71–2.13), respectively. (ii) Estimates

remained consistent after common-support trimming. (iii) Overlap weighting yielded an HR of 1.15 (95% CI 0.66–2.00). (iv) Propensity-score matching on the logit of the PS (caliper $= 0.2 \times$ SD) identified 13 1:1 pairs via nearest-neighbour matching without replacement (15 pairs with replacement); 1:2 variable-ratio matching produced 13 matched treated patients; and optimal 1:1 matching yielded 15 pairs. Pair-stratified Cox models with robust variance were used throughout.

Stabilized IPTW was retained as the primary estimator for between-regimen comparisons because it maximized sample retention and balance. Sensitivity analyses using truncation, trimming, overlap weighting, and multiple matching specifications produced qualitatively similar findings, supporting the robustness of the main results.

2 SUPPLEMENTARY FIGURES

Table S1. Anlotinib dosing characteristics in the ICI+Chemo+Anlotinib arm

Variable	Category	ICI+Chemo+Anlotinib (n = 17), n (%)
Starting dose	12 mg once daily	10 (58.8)
	10 mg once daily	7 (41.2)
Early dose reduction within 2 cycles	Yes	1 (5.9)
	No	16 (94.1)

Percentages are calculated within the ICI+Chemo+Anlotinib arm (n = 17). Early dose reduction was defined as any reduction from the starting dose within the first two treatment cycles.

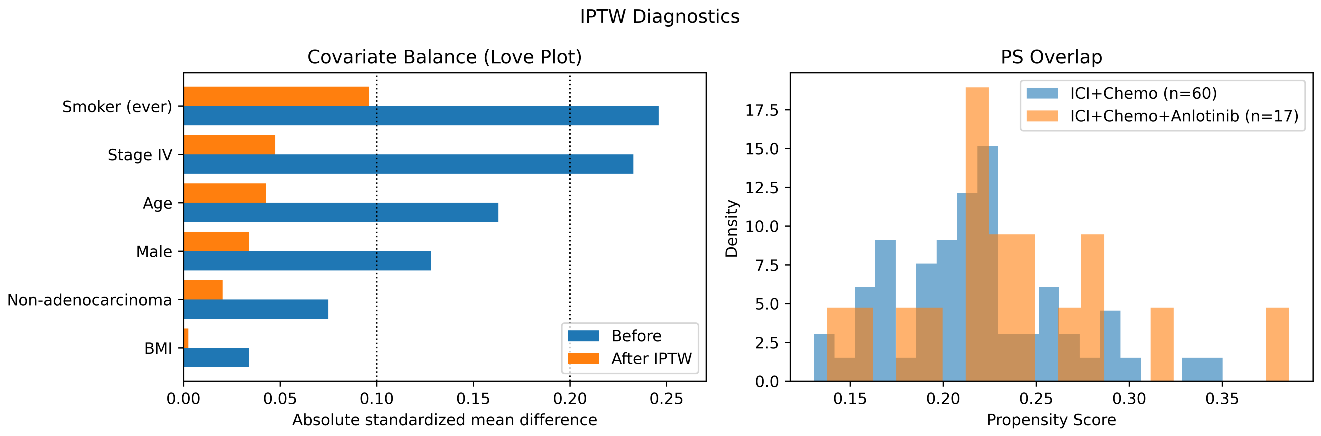

Figure S1. Figure S1. IPTW diagnostics. Left: covariate balance (Love plot) showing absolute standardized mean differences (SMDs) before and after weighting for prespecified covariates (age, sex, BMI, smoker ever, histology [non-adenocarcinoma vs. adenocarcinoma], and stage [IV vs. III]); vertical reference lines at 0.10 and 0.15 denote commonly used thresholds for acceptable balance. Right: propensity-score overlap between treatment arms (ICI+Chemo vs. ICI+Chemo+Anlotinib). All weighted SMDs were < 0.15 (most < 0.10), indicating adequate balance with good PS overlap.

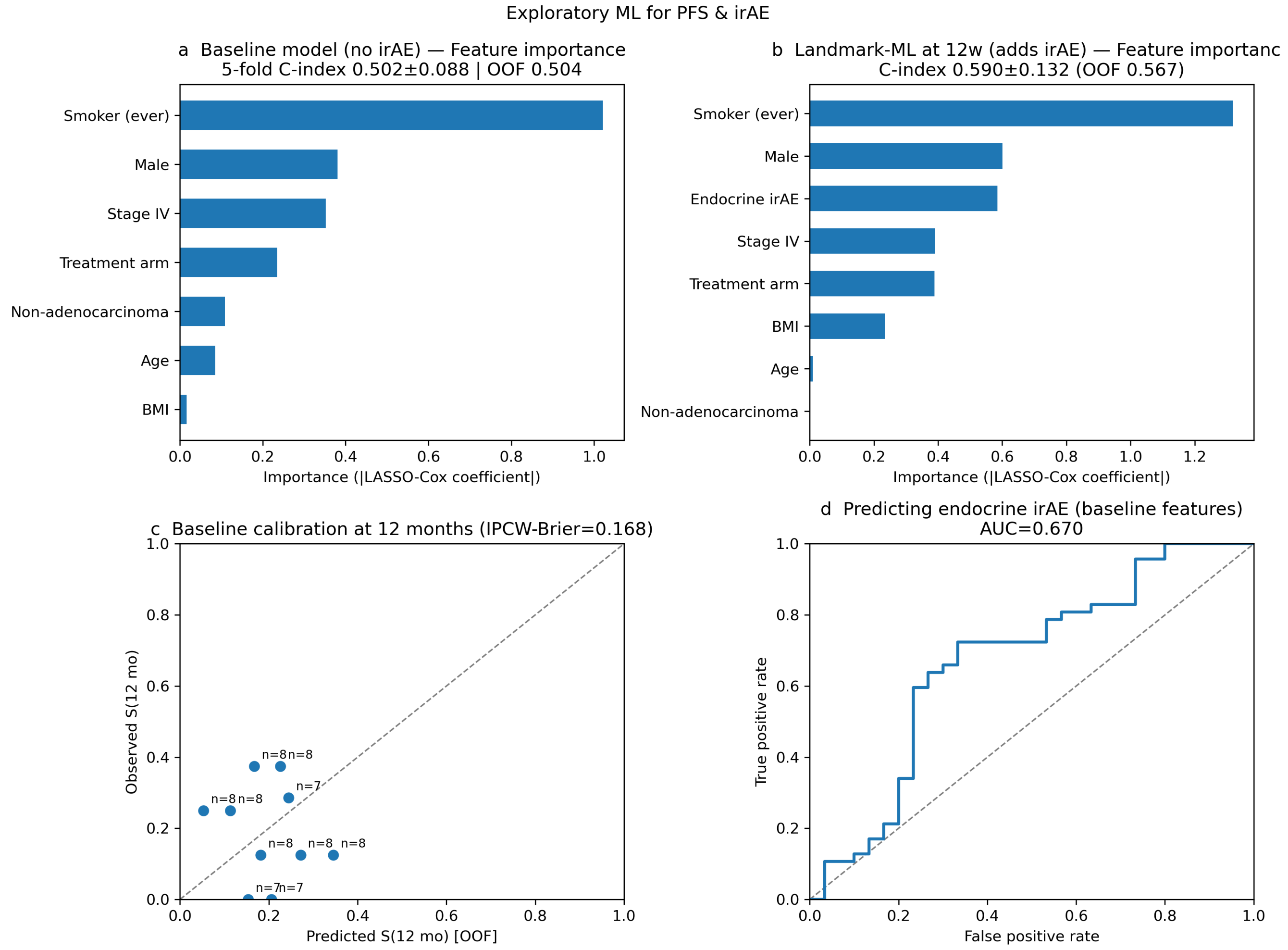

Figure S2. Figure S2. Exploratory machine-learning analyses for PFS and endocrine irAEs. (a) Baseline model without irAE status: LASSO–Cox feature importance; 5-fold C-index = 0.502 ± 0.088, out-of-fold (OOF) C-index = 0.504. (b) 12-week landmark model including irAE status: feature importance; cross-validated C-index = 0.590 ± 0.132, OOF C-index = 0.567. (c) Calibration of the baseline model at 12 months (IPCW-Brier = 0.168). (d) L1-penalized logistic regression predicting endocrine irAE from baseline features (OOF AUC = 0.670).

3 SUPPLEMENTARY TABLES

Table S2. Assay-specific thresholds and reference intervals used for endocrine irAE definitions (Zhoushan Branch, Ruijin Hospital)

Analyte	Unit	Lower limit (LLN)	Upper limit (ULN)
TSH	µIU/mL	0.55	4.78
Free T4 (FT4)	pmol/L	11.5	22.7
Free T3 (FT3)	pmol/L	3.5	6.5
Total T4 (T4)	nmol/L	58.1	140.6
Total T3 (T3)	nmol/L	0.92	2.79
Morning cortisol (08:00–09:00)	µg/dL	4.30	22.40
ACTH	pg/mL	7.20	63.30
Cosyntropin peak cortisol[a]	µg/dL	18.0	–

Units and reference intervals are from the institutional laboratory. [a]For dynamic testing with 250 µg cosyntropin, a peak cortisol < 18.0 µg/dL was considered subnormal according to local practice.

Table S3. Baseline characteristics by endocrine irAE status within each treatment arm

Characteristic	**ICI+Chemo+Anlotinib** (n=17)			**ICI+Chemo** (n=60)		
	**with irAE** (n=11)	**without irAE** (n=6)	**P**	**with irAE** (n=16)	**without irAE** (n=44)	**P**
**Age, median (range)**						
Years	64 (54–73)	63 (49–78)	1.0	68 (49–79)	63 (49–79)	0.25
**Sex, n (%)**						
Female	3 (27.3%)	1 (16.7%)	1.0	9 (25.0%)	2 (8.3%)	0.17
Male	8 (72.7%)	5 (83.3%)		27 (75.0%)	22 (91.7%)	
**Smoking status, n (%)**						
Never	1 (9.1%)	1 (16.7%)	1.0	2 (5.6%)	1 (4.2%)	1.0
Current / Former	10 (90.9%)	5 (83.3%)		34 (94.4%)	23 (95.8%)	
**Histological features, n (%)**						
Adenocarcinoma	7 (63.6%)	1 (16.7%)	0.13	16 (44.4%)	10 (41.7%)	1.0
Non-adenocarcinoma	4 (36.4%)	5 (83.3%)		20 (55.6%)	14 (58.3%)	
**Stage, n (%)**						
III	3 (27.3%)	3 (50.0%)	0.60	10 (27.8%)	18 (75.0%)	0.00
IV	8 (72.7%)	3 (50.0%)		26 (72.2%)	6 (25.0%)	

Table S4. Baseline PD-L1 tumour proportion score (TPS) and oncogenic driver status.

Variable	Overall (N=77)	ICI+Chemo+Anlotinib (n=17)	ICI+Chemo (n=60)
**PD-L1 TPS**			
$<1\%$	1 (1.3%)	0 (0.0%)	1 (1.7%)
1–49%	4 (5.2%)	1 (5.9%)	3 (5.0%)
$\geq 50\%$	3 (3.9%)	0 (0.0%)	3 (5.0%)
Unknown / not tested	69 (89.6%)	16 (94.1%)	53 (88.3%)
**Oncogenic driver status**			
Positive (EGFR/ALK/ROS1/other)	16 (20.8%)	5 (29.4%)	11 (18.3%)
Negative	17 (22.1%)	3 (17.6%)	14 (23.3%)
Unknown / not genotyped	44 (57.1%)	9 (52.9%)	35 (58.3%)

Percentages are calculated within each column (treatment group). PD-L1 tumour proportion score (TPS) was available in 8/77 patients (10.4%); "Unknown / not tested" indicates that PD-L1 was not assessed or that results were not retrievable from the record. Driver-positive status indicates the presence of an oncogenic alteration in EGFR, ALK, ROS1 or another reported driver. "Unknown / not genotyped" indicates that molecular testing was not performed or not documented.
